# Renal epidermoid cyst mimicking renal tuberculous abscess: a case report

**DOI:** 10.3389/fmed.2025.1632764

**Published:** 2025-07-22

**Authors:** Jian Gao, Huijiu Luo, Han Zhu, Zhengdao Liu, Mingzhou Li, Yuzhu Chen, Shiyu Wang, Chao Zhou, Zhenhao Li, Guobiao Liang, Shulian Chen

**Affiliations:** Department of Urology, Affiliated Hospital of Zunyi Medical University, Zunyi, Guizhou, China

**Keywords:** renal epidermoid cyst, renal tuberculous abscess, cystic renal lesion, pseudoaneurysm, partial nephrectomy, empirical anti-tuberculosis therapy, case report

## Abstract

Renal epidermoid cysts (RECs) are exceedingly rare benign cystic lesions, with only 15 histologically confirmed cases reported worldwide to date. Due to their non-specific clinical and radiological features, they are often misdiagnosed preoperatively as infectious or neoplastic conditions. Here, we report a 25-year-old man in whom a complex renal cyst was incidentally identified during a routine health examination. Retrospectively, the patient reported mild urinary frequency and low-grade fever. Imaging suggested a non-enhancing heterogeneous cyst in the lower pole of the right kidney. Laparoscopic partial nephrectomy was performed, revealing abundant yellow-white caseating material intraoperatively, prompting empirical anti-tuberculosis therapy in the context of regional endemicity. However, histopathological analysis confirmed a diagnosis of RECs, and anti-tuberculous treatment was subsequently withdrawn. On postoperative day 5, the patient developed gross hematuria due to a renal artery pseudoaneurysm, which was successfully managed with selective arterial embolization. This case highlights the diagnostic challenges posed by atypical cystic renal lesions and underscores the importance of integrating imaging, intraoperative findings, and histopathology. Including RECs in the differential diagnosis may prevent unnecessary antituberculous therapy and overtreatment.

## Introduction

Renal epidermoid cysts (RECs) are an exceptionally rare, benign, non-neoplastic cystic lesion of unknown origin. According to the 2022 WHO Classification of Tumours of the Urinary System and Male Genital Organs, it is classified under “non-neoplastic cysts of uncertain etiology” (1). As of June 2025, only 15 histologically confirmed cases have been reported in the literature, all of which were misdiagnosed preoperatively due to their non-specific clinical symptoms and radiological findings. RECs typically present as a solitary cystic mass lined with keratinizing squamous epithelium, with the lumen filled with laminated keratinous debris (2). Its imaging features, however, are highly variable and often mimic those of renal cell carcinoma (RCC) or renal abscesses, making accurate preoperative identification difficult. On ultrasound or computed tomography (CT), RECs have been described as large, heterogeneous, complex cystic masses with thickened walls and occasional calcification. In our literature review (Table 1), 7 out of the 15 reported cases were preoperatively mistaken for RCC or other renal tumors (2–8), and 9 patients (60%) presented with non-specific symptoms such as flank pain or hematuria (2–4, 7–12).

**Table 1:** Cases reported in the literature.

Author	Time	Sex	Age	Flank pain	Haematuria	Radiologic presentation	Presence of calcification	Association with stones	Preoperative impression	Surgical treatment
Krogdahl et al. (9)	1979	M	67	Yes	No	Unknown	Yes	No	Old renal	Partial nephrectomy
									Tuberculosis	
Duprat et al. (16)	1986	M	4	No	Yes	Renal mass	Yes	No	Renal teratoma	Radical nephrectomy
Emtage and Allen (3)	1994	F	74	Yes	No	Renal mass	No	No	Renal cell carcinoma	Radical nephrectomy
Lim and Kim (4)	2003	M	51	Yes	Yes	Renal mass	Yes	Renal stone	Cystic renal mass	Simple nephrectomy
Gokce and Kaya (2)	2003	F	55	Yes	No	Renal pelvis mass	Yes	Renal stone	Renal pelvis tumor	radical
										nephroureterectomy
Abdou and Asaad (15)	2010	M	67	No	No	Renal mass	No	No	End stage renal disease	Simple nephrectomy
Dadali et al. (10)	2010	F	48	Yes	Yes	Atrophic kidney	No	No	Chronic pyelonephritis	Simple nephrectomy
Bauer et al. (13)	2010	F	68	Yes	No	Renal pelvis mass	Yes	Renal stone	Epidermal cyst through	Partial nephrectomy
									Ureterorenoscopic biopsy	
Desai et al. (5)	2011	M	74	No	Yes	Renal mass	Yes	Renal stone	Renal cell carcinoma	Radical nephrectomy
Go et al. (17)	2012	F	73	No	No	Renal pelvis mass	No	No	Renal pelvis tumor	Nephroureterectomy
Cabrales et al. (11)	2016	F	45	Yes	No	Renal pelvis mass	Yes	Renal stone	Renal stone	PCNL
Pradhan et al. (6)	2017	F	62	No	No	Renal mass	No	Renal stone	Renal cell carcinoma	Radical nephrectomy
Barrios Barreto et al. (7)	2021	M	56	Yes	Yes	Renal mass	Yes	Renal stone	Renal cell carcinoma	Partial nephrectomy
Ido et al. (8)	2023	F	45	Yes	Yes	Renal mass	NO	No	Renal cell carcinoma	Simple nephrectomy
Khorraminejad-Shirazi et al. (12)	2024	M	64	Yes	Yes	Renal mass	Yes	Renal stone	Renal cell carcinoma	Radical nephrectomy
Present case	2024	M	25	No	No	Renal mass	No	No	Cystic renal mass	Partial nephrectomy

Notably, among all reported cases, only the one described by Bauer et al. suggested a diagnosis of epidermoid cyst prior to surgery based on ureteroscopic biopsy, although no definitive radiological diagnosis was established (13). Although the postoperative prognosis for RECs is generally favorable, the lack of pathognomonic imaging signs underscores the diagnostic challenge.

In the present case, imaging revealed a complex cyst in the right kidney, and intraoperative findings suggested an infectious etiology due to the presence of thick, yellow-white caseous material—mimicking renal tuberculous abscess (RTA). The final diagnosis of RECs was established only after histopathological examination. Through this case, we aim to raise clinical awareness of this rare entity and emphasize the importance of including RECs in the differential diagnosis of complex renal cysts, particularly in regions where renal tuberculosis is endemic.

## Case presentation

A 25-year-old unmarried man with no prior history of renal disease, nephrolithiasis, tuberculosis, urinary tract infections, or abdominal surgery was found to have a right renal mass during a routine pre-employment health screening. He denied any family history of hereditary kidney disease, genitourinary malignancies, or consanguinity. There were no known psychosocial stressors, and he reported no history of smoking, alcohol consumption, or illicit drug use. Additionally, there was no evidence of any underlying genetic syndromes.

Renal ultrasonography revealed a well-defined, 6.5 × 5.1 cm isoechoic lesion with heterogeneous internal echogenicity and no detectable intralesional blood flow at the lower pole of the right kidney. No hydronephrosis was observed, and the contralateral kidney appeared normal.

Retrospectively, the patient reported 1 week of mild urinary frequency and intermittent low-grade fever (maximum temperature 37.8°C), though he denied flank pain, hematuria, weight loss, or any other constitutional symptoms at the time of detection.

There was no prior history of nephrolithiasis, tuberculosis, renal surgery, or urinary tract infections. Physical examination showed right costovertebral angle (CVA) tenderness, likely due to capsular distension from the cystic lesion; there was no tenderness along the bilateral ureters, and the remainder of the examination was unremarkable. Laboratory tests revealed leukocytosis (WBC 10.29 × 10^9^/L; neutrophils 8.64 × 10^9^/L; neutrophil percentage 84%), elevated serum creatinine (135 μmol/L), reduced eGFR (64.45 mL/min/1.73 m^2^), and increased high-sensitivity C-reactive protein (34.571 mg/L). No previous creatinine or eGFR records were available for comparison; therefore, the mildly elevated creatinine value observed may reflect individual physiological variation, particularly increased muscle mass in young adult males, rather than impaired renal function.

Urinalysis showed yellow urine with significant pyuria (++). Smears and cultures for acid-fast bacilli (AFB) in urine and sputum were negative. Additional tuberculosis screening, including serum TB-IgG and PPD skin tests, also returned negative.

**Figure 1:**
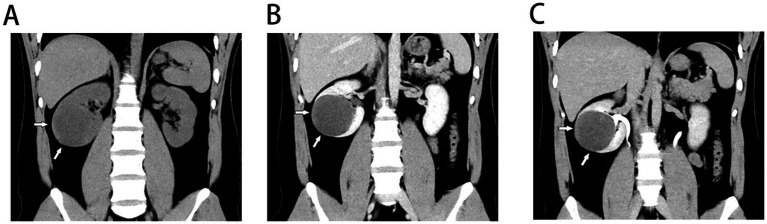
Preoperative CT scan: **(A)** Non-contrast coronal CT image revealed a well-defined mass measuring 7 cm in the right kidney with slightly higher density around the margin. **(B)** Arterial phase image of contrast-enhanced CT showed no notable enhancement within the lesion. **(C)** Excretory phase image of contrast-enhanced suggested no contrast agent retention in the cyst and no discernible connection to the pelvis or calyces.

Repeat ultrasonography confirmed a well-circumscribed cystic mass in the lower pole of the right kidney, with no internal vascularity. Non-contrast and contrast-enhanced abdominal CT with 3D reconstruction revealed a 7.0-cm spherical, well-marginated cystic lesion with heterogeneous internal density and no appreciable enhancement (Figures 1A,B). There was no communication with the pelvicalyceal system on excretory phase imaging (Figure 1C). These findings, along with mild inflammatory markers, led to a provisional diagnosis of a complex renal cyst, possibly infectious or a rare benign cystic tumor. Given the Bosniak III features and the limitations of percutaneous biopsy in keratin-containing lesions, diagnostic and therapeutic laparoscopic partial nephrectomy was performed. Intraoperatively, the lesion appeared cystic, soft, and encapsulated, with the cavity filled with abundant yellow-white caseating material. Given the lesion’s appearance and the regional endemicity of tuberculosis, an intraoperative suspicion of an RTA arose. Although all preoperative tuberculosis tests—including serum TB-IgG, purified protein derivative (PPD) skin test, and AFB smears of urine and sputum—were negative, empirical anti-tuberculosis treatment with isoniazid, rifampin, pyrazinamide, and ethambutol was initiated postoperatively.

Concurrently, broad-spectrum antibiotics (ceftriaxone and metronidazole) were administered perioperatively to cover potential bacterial superinfection. This dual therapeutic approach reflected both the intraoperative impression of possible tuberculous infection and the uncertainty surrounding the preoperative diagnosis.

**Figure 2:**
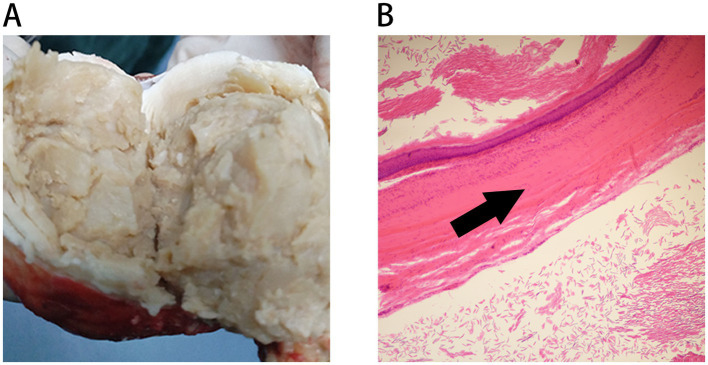
Macroscopic and microscopic pictures of the specimen: **(A)** Gross appearance disclosed a well-circumscribed solid mass with friable whitish to yellowish amorphous contents. **(B)** Microscopy revealed the cyst was lined by stratified squamous epithelium (indicated by black arrow) and was filled by lamellated keratin (upper left corner; H&E; ×40).

Intraoperatively, the lesion was located at the lower pole of the right kidney, presenting as a soft, encapsulated, cystic mass with intact walls. The resected mass measured approximately 7.0 × 6.0 × 5.5 cm. Upon incision, the cyst cavity was filled with abundant yellow-white caseating material (Figure 2A), with no evidence of hair, sebaceous glands, calcification, or malignancy. In the setting of regional tuberculosis endemicity and intraoperative suspicion for an RTA, empirical anti-tuberculous therapy (isoniazid, rifampin, pyrazinamide, and ethambutol) was initiated. Broad-spectrum antibiotics (ceftriaxone and metronidazole) were also administered perioperatively.

Histopathological examination of the specimen demonstrated a cyst wall lined by stratified squamous epithelium, with the lumen containing laminated keratinous debris but no Langhans giant cells or granulomatous inflammation (Figure 2B). Ziehl–Neelsen staining and PCR for *Mycobacterium tuberculosis* (TB-PCR) were both negative. Immunohistochemistry was positive for CK5/6, confirming squamous epithelial origin. These findings established the final diagnosis of RECs. Empirical anti-tuberculosis treatment was discontinued on postoperative day 3.

**Figure 3:**
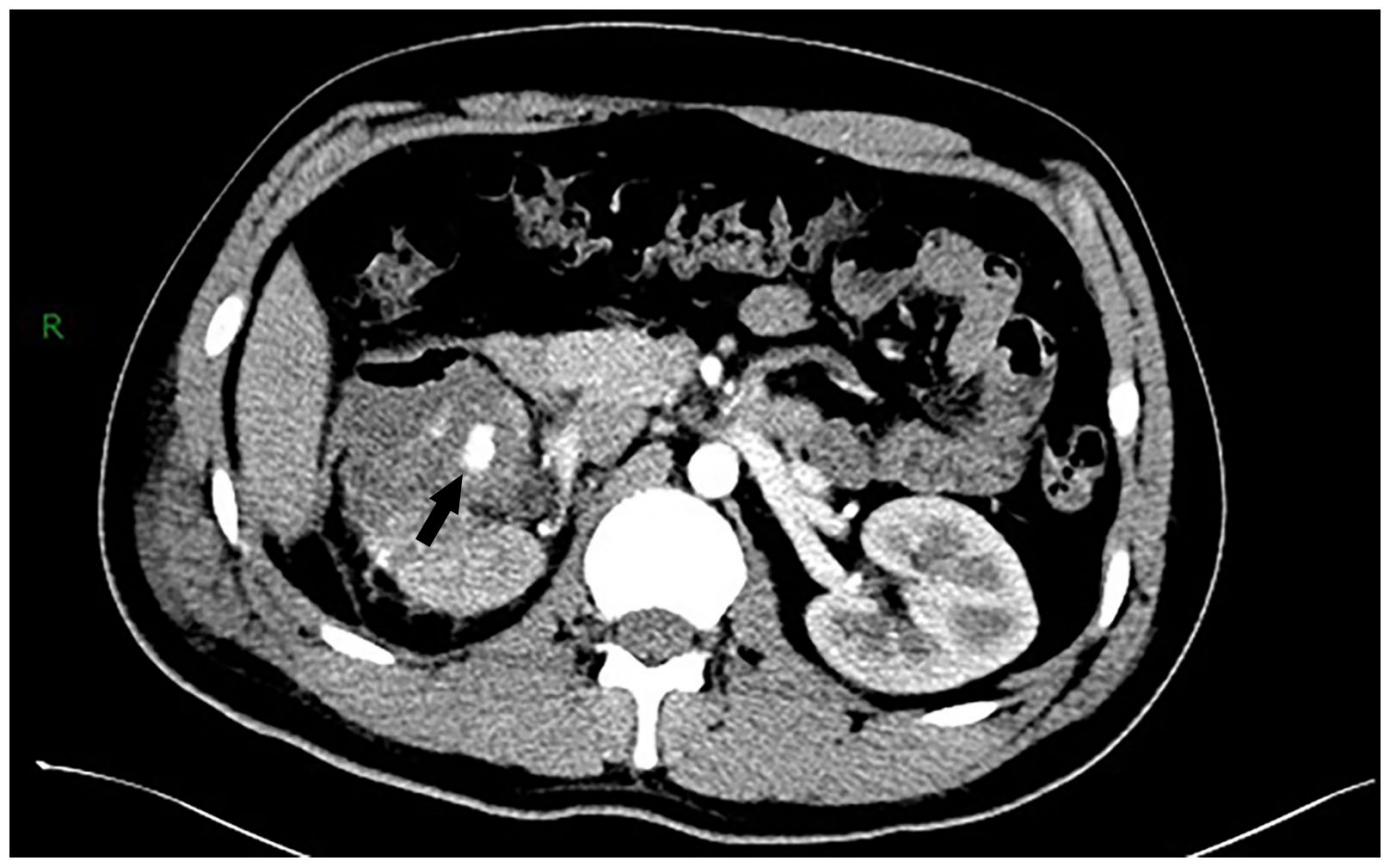
Postoperative contrast-enhanced CT disclosed an enhancement lesion in the right kidney, suspicious for a RAP (indicated by the black arrow).

**Figure 4:**
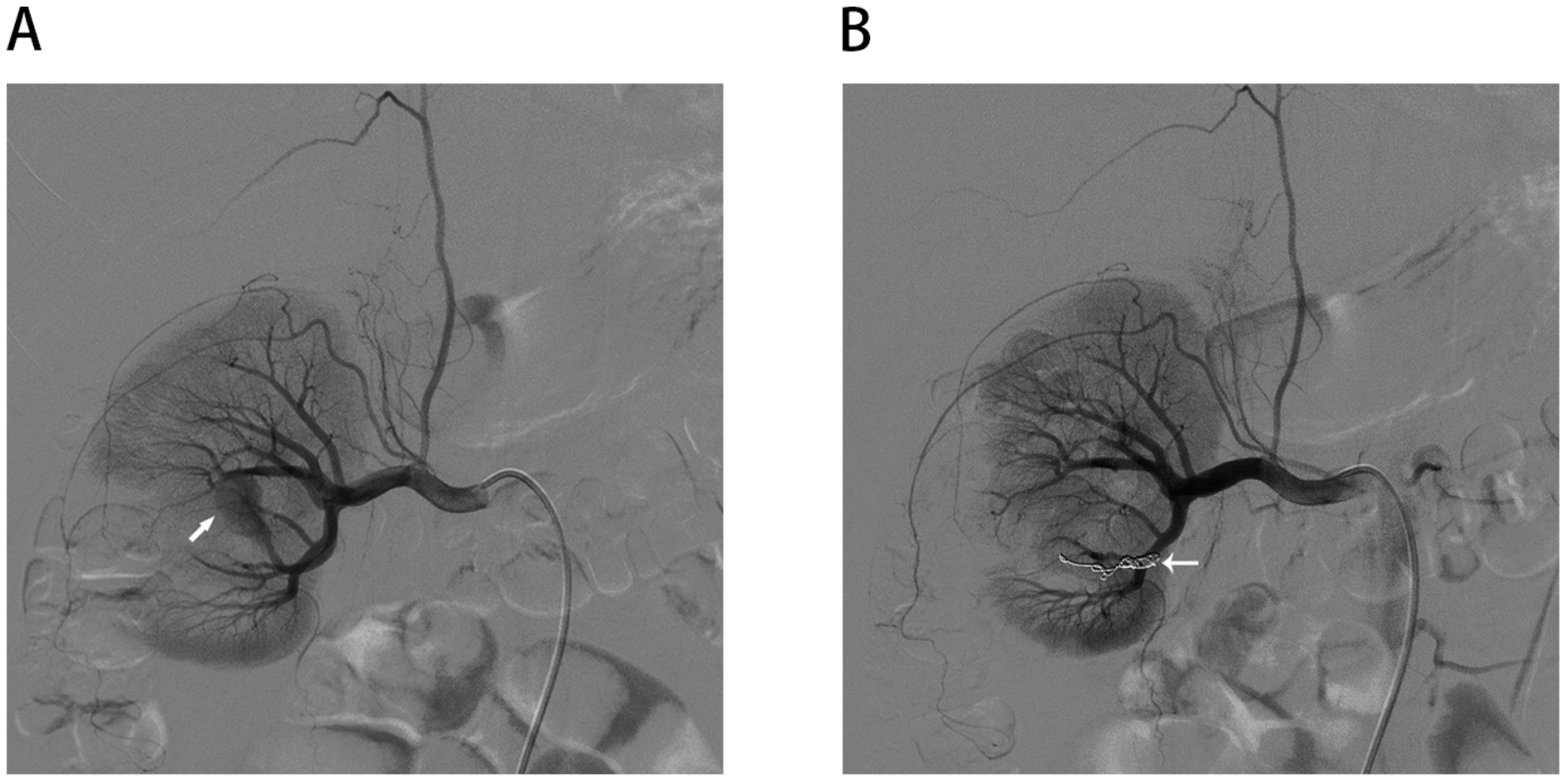
Postoperative angiograms. **(A)** Renal arterial angiogram revealed the formation of RAP. **(B)** No extravasation of radiographic contrast was found after selective angioembolization.

On postoperative day 5, the patient developed gross hematuria following ambulation. Initial management involved bed rest and hemostatic agents. However, persistent bleeding led to contrast-enhanced CT imaging, which demonstrated a focal hyperenhancing lesion at the surgical site, suggesting a renal artery pseudoaneurysm (Figure 3). On postoperative day 19, digital subtraction angiography confirmed a pseudoaneurysm arising from a lower pole segmental artery. Superselective microcatheter-guided embolization was performed successfully (Figures 4A,B). The hematuria resolved, and no recurrence was observed during 6 months of follow-up. A detailed chronological summary of diagnostic, therapeutic, and follow-up milestones is provided in Table 2.

**Table 2:** Timeline of clinical events during diagnostic and therapeutic management.

DAY	Event
0	Routine health check, incidental renal mass discovered
2	CT imaging confirms complex cystic lesion
3	Admitted for further evaluation and preoperative workup
5	Underwent laparoscopic partial nephrectomy
6	Initiated empirical anti-TB therapy due to intraoperative findings
7	Started ceftriaxone and metronidazole postoperatively
9	Anti-TB therapy discontinued after histopathology confirmed REC
10	Postoperative gross hematuria noted
24	DSA confirmed pseudoaneurysm; embolization performed
180	6-month follow-up: no recurrence, stable renal function

A chronological summary of key clinical events from initial detection of the renal mass during a routine health screening to 6-month postoperative follow-up. CT, computed tomography; TB, tuberculosis; REC, renal epidermoid cyst; DSA, digital subtraction angiography.

## Patient perspective

The patient initially experienced significant anxiety due to the incidental discovery of a renal mass, particularly given concerns about a possible malignancy or tuberculosis. Following a comprehensive diagnostic evaluation and confirmation of a benign lesion, he expressed a strong sense of relief and gratitude. He reported satisfaction with the multidisciplinary care he received, including timely surgical intervention and resolution of the postoperative complication. Furthermore, he emphasized the importance of patient education regarding rare renal conditions and the need for structured follow-up to ensure long-term health and peace of mind.

## Discussion

In this case, a right-sided cystic renal lesion was incidentally discovered in a young male patient during routine health screening. Although he retrospectively reported urinary frequency and low-grade fever, the clinical presentation remained non-specific. Imaging studies revealed a well-defined cystic mass with heterogeneous internal density, and laboratory tests showed mild inflammatory markers. The preoperative differential diagnosis included a complex renal cyst, infectious cyst, or a benign cystic tumor.

Given the lesion’s classification as Bosniak III, which carries a substantial risk of malignancy (approximately 50%), and the limitations of percutaneous biopsy in keratin-containing cysts, surgical excision was deemed both diagnostic and therapeutic (14).

Intraoperatively, the lesion appeared thick-walled and was filled with yellow-white caseating material, raising suspicion for RTA. Given the high tuberculosis burden in the region and intraoperative findings, empirical anti-tuberculosis therapy was initiated. Postoperative histopathology confirmed a diagnosis of RECs, leading to the discontinuation of anti-tuberculosis treatment. This case highlights the importance of correlating preoperative imaging with intraoperative morphology and histopathological confirmation to avoid misdiagnosis and overtreatment.

RECs are an extremely rare, benign, non-neoplastic cystic lesion, with only 15 cases reported to date (2–13, 15–17). Radiologically, RECs typically appear as solitary, well-demarcated cystic masses with heterogeneous internal contents. High attenuation areas on CT or hyperechogenicity on ultrasound are believed to reflect intraluminal keratinous debris. In contrast-enhanced imaging, RECs typically lack significant enhancement (7, 8, 12). These imaging characteristics closely resemble those of early-stage RTAs, which may also present as complex cystic masses with ring-like enhancement, intracystic sediment, or debris (18). However, these features are non-specific, and both conditions may manifest as low-attenuation, non-enhancing lesions. Clinically, both entities may present with vague symptoms such as back pain, fever, or hematuria. Consequently, the preoperative differentiation between RECs and RTA is particularly challenging—especially in endemic areas—and relies heavily on histopathological examination.

Notably, in this case, comprehensive tuberculosis screening, including T-SPOT, PPD, acid-fast staining, and TB-PCR, was negative, underscoring the diagnostic limitations of imaging and laboratory tests alone. As shown in Table 3, RECs and RTA differ significantly in pathogenesis, treatment, and histopathological features, yet often converge in preoperative appearance. Thus, awareness of RECs as a differential diagnosis is critical to avoid unnecessary medical therapy, including anti-tuberculous regimens.

**Table 3:** Comparison of clinical, radiological, pathological, and therapeutic features between renal epidermoid cyst and renal tuberculous abscess.

Feature	Renal Epidermoid Cyst (REC)	Renal Tuberculous Abscess (RTA)
Epidemiology	Extremely rare; ~15 cases reported worldwide	More common in TB-endemic areas
Etiology	Non-neoplastic cyst of unknown origin (WHO 2022)	*Mycobacterium tuberculosis* infection
Clinical presentation	Flank pain, hematuria, or asymptomatic; low-grade fever occasionally	Urinary frequency, low-grade fever, weight loss, night sweats
Imaging features	Large, well-defined cystic mass with heterogeneous density; no enhancement; mimics RCC or complex cyst	Low-attenuation cyst with rim enhancement; calcification, cortical scarring, collecting system distortion possible
Ultrasound findings	Mixed echogenicity; no vascular signal	Hypoechoic lesion with debris; may show ring enhancement
CT findings	No contrast enhancement; possible wall calcification; keratinous debris causes high attenuation	Rim enhancement on contrast CT; possible parenchymal scarring or deformation
Laboratory results	Mild leukocytosis; AFB and TB-PCR negative	Sterile pyuria, positive TB tests (T-SPOT, IGRA, PPD); possible AFB+/TB-PCR+
Intraoperative appearance	Yellow-white caseous contents; no sebum, no granulomas	Caseous necrosis, granulomas, fibrosis
Histopathology	Squamous epithelium, laminated keratin, CK5/6+; no granulomas	Granulomatous inflammation, caseating necrosis, Langhans cells
Treatment	Partial or radical nephrectomy	Anti-TB chemotherapy (RIPE); drainage or nephrectomy in severe cases
Prognosis	Excellent with complete resection	Variable; depends on TB control and parenchymal damage

Intraoperative assessment further supports the diagnostic distinction. The lesion was sharply demarcated with classic cystic morphology, filled with keratinous material but lacking features typical of dermoid cysts, teratomas, or infectious granulomas. Histological analysis revealed stratified squamous epithelium lining and laminated keratinous debris. Immunohistochemistry was positive for CK5/6, supporting squamous epithelial origin. Other differential diagnoses—including mature teratoma, dermoid cyst, renal tuberculosis, and xanthogranulomatous pyelonephritis—were ruled out by the absence of hair, sebaceous glands, and granulomatous inflammation (19, 20).

On postoperative day 5, the patient developed gross hematuria. Imaging revealed a renal artery pseudoaneurysm, a known vascular complication following partial nephrectomy, particularly in cases involving deep cortical resection or proximity to the renal hilum. The pseudoaneurysm was successfully treated with superselective arterial embolization, and the patient recovered without further incident. This complication is not specific to RECs but underscores the importance of recognizing delayed postoperative vascular events (21).

In summary, this case reinforces several clinical insights. First, in patients with atypical cystic renal lesions and intraoperative “caseous” material, clinicians should consider RECs in the differential—even in tuberculosis-endemic regions. Second, the diagnosis should integrate epidemiologic history, imaging, intraoperative observations, and definitive histopathology. Third, the initiation of empirical anti-tuberculosis therapy should be reserved for cases with compelling microbiologic or molecular evidence. Finally, awareness of potential vascular complications such as pseudoaneurysm is essential for timely postoperative management. Broader recognition of RECs can prevent unnecessary nephrectomy and antituberculous treatment, ultimately improving patient outcomes.

Despite the favorable outcome in this case, several limitations merit discussion. First, magnetic resonance imaging (MRI), which may have provided better soft-tissue contrast and additional preoperative clues, was not performed. Second, the follow-up duration was limited to 6 months, which may be insufficient to assess long-term recurrence or complications. Finally, due to the rarity of RECs, standardized diagnostic criteria are lacking. Further case series or multicenter studies are needed to better characterize its clinical course and imaging features, and to guide evidence-based management.

This case demonstrates the diagnostic ambiguity of renal epidermoid cysts, especially in TB-endemic regions. A key strength of this report is the documentation of intraoperative misdiagnosis and its reversal following histopathological confirmation. However, limitations include the lack of preoperative MRI and the absence of long-term follow-up beyond 6 months. Compared to the limited existing literature, this case adds valuable insight into the risk of overtreatment and reinforces the importance of histological verification in atypical renal cysts.

## Strengths and limitations

A limitation of this case report is the single-patient design without longitudinal imaging comparison beyond 6 months. Moreover, preoperative MRI was not performed, which may have provided additional diagnostic clarity. Nonetheless, this report contributes valuable clinical insight into the diagnostic ambiguity of rare renal cystic lesions in tuberculosis-endemic settings. Future multicenter studies are warranted to validate imaging criteria and support diagnostic standardization of RECs.

## Conclusion

RECs are an exceptionally rare, benign, non-neoplastic cystic lesion that may closely mimic both infectious and neoplastic renal conditions in clinical and radiological presentations. This case illustrates the diagnostic challenges posed by atypical cystic renal masses, particularly in tuberculosis-endemic regions, where imaging and intraoperative findings may resemble those of renal tuberculous abscesses. Accurate diagnosis requires a comprehensive approach that integrates imaging, intraoperative assessment, and definitive histopathological analysis.

Additionally, clinicians should remain alert to delayed postoperative vascular complications—such as renal artery pseudoaneurysm following partial nephrectomy—which necessitate timely recognition and interventional management. Including RECs in the differential diagnosis of complex renal cysts can help avoid unnecessary treatment, improve diagnostic accuracy, and ultimately enhance patient outcomes.

## Data Availability

The original contributions presented in the study are included in the article/supplementary material, further inquiries can be directed to the corresponding authors.
